# Elevated serum IL-6 levels predict treatment interruption in patients with moderate to severe psoriasis: a 6-year real-world cohort study^☆^

**DOI:** 10.1016/j.abd.2023.03.002

**Published:** 2023-08-25

**Authors:** Natália Ribeiro de Magalhães Alves, Patrícia Shu Kurizky, Licia Maria Henrique da Mota, Cleandro Pires de Albuquerque, Juliana Tomaz Esper, Aridne Souza Costa Campos, Vitoria Pereira Reis, Henrique Metzker Ferro, Natalia Gil-Jaramillo, Joaquim Pedro Brito-de-Sousa, Luana Cabral Leão Leal, Otávio de Toledo Nóbrega, Carla Nunes de Araújo, Agenor de Castro Moreira dos Santos Júnior, Gladys Aires Martins, Olindo Assis Martins Filho, Ciro Martins Gomes

**Affiliations:** a Programa de Pós-Graduação de Ciências Médicas, Faculdade de Medicina, Universidade de Brasília, Brasília, DF, Brazil; b Serviço de Dermatologia, Hospital Universitário de Brasília, Brasília, DF, Brazil; c Serviço de Reumatologia, Hospital Universitário de Brasília, Brasília, DF, Brazil; d Programa de Pós-Graduação em Patologia Molecular, Faculdade de Medicina, Universidade de Brasília, Brasília, DF, Brazil; e Laboratório de Interação Patógeno-Hospedeiro, Instituto de Ciências Biológicas, Universidade de Brasília, Brasília, DF, Brazil; f Instituto de Ciências Biomédicas, Universidade Federal de Uberlândia, Uberlândia, MG, Brazil; g Laboratório Central de Saúde Pública do Distrito Federal, Secretaria de Saúde do Distrito Federal, Brasília, DF, Brazil; h Instituto René Rachou, Fundação Oswaldo Cruz, Fiocruz Minas, Belo Horizonte, MG, Brazil

**Keywords:** Allergy and immunology, Autoimmune disease, Biomarkers, Immunosuppression therapy, Psoriasis

## Abstract

**Background:**

Real-world, primary data on the treatment of psoriasis are scarce, especially concerning the role of soluble biomarkers as outcome predictors.

**Objective:**

The authors evaluated the utility of Th1/Th17 serum cytokines along with clinical characteristics as predictors of drug survival in the treatment of psoriasis.

**Methods:**

The authors consecutively included participants with moderate to severe psoriasis who were followed up for 6 years. Baseline interferon-α, tumor necrosis factor-α, and interleukin (IL)-2, IL-4, IL-6, IL-10, and IL-17A were measured using a cytometric bead array; clinical data were assessed. The authors calculated hazard ratios (HRs) for drug survival using a Cox proportional hazards model.

**Results:**

The authors included 262 patients, most of whom used systemic immunosuppressants or biologics. In the multivariate model, poor quality of life measured by the Dermatology Life Quality Index (HR = 1.04; 95% CI 1.01‒1.07; p = 0.012) and elevated baseline IL-6 (HR = 1.99; 95% CI 1.29‒3.08; p = 0.002) were associated with treatment interruption.

**Study limitations:**

The main limitation of any cohort study is the presence of confounders that could not be detected in clinical evaluation.

**Conclusions:**

Poor quality of life and elevated baseline serum IL-6 level predicted treatment interruption in patients with moderate to severe psoriasis. Although IL-6 is not the most important mediator of the inflammatory pathway in the skin environment, it is an interesting biomarker candidate for predicting psoriasis treatment response.

## Introduction

Psoriasis is a chronic disease that affects between 1% and 3% of the global population.^1^, ^2^ The revolution in the development of new biologic drugs and small molecules is supported by high-quality clinical trials.^3^, ^4^ Those medications act through modulated blocking of cytokines involved in the pathogenesis of psoriasis, reducing the occurrence of adverse events and achieving better results in comparison to classic immunosuppressants.^5^, ^6^ Since new medications are being constantly released in the market, the necessity of additional data targeting the pharmacovigilance of immunosuppressors for the treatment of psoriasis is being constantly emphasized.^7^, ^8^ The number of active registries for monitoring adverse events is still insufficient.^9^, ^10^

Psoriasis is a disease that affects the skin and other organs through systemic inflammation.^11^, ^12^, ^13^ The disease has also a significant impact on a patient's quality of life.^14^ In general, pharmacovigilance data is scarce in developing countries.^15^ The Brazilian Unified Health System (SUS) is responsible for the provision of healthcare and pharmaceutical assistance to over 200 million people.^16^ Therefore, the Brazilian SUS constitutes a privileged scenario for pharmacovigilance.^17^ The national protocol regarding the sequence of medications used in psoriasis is not different from most international recommendations.^18^

The authors aimed to evaluate drug survival in moderate to severe psoriasis. The authors also aimed to evaluate whether serum cytokines can be used to predict psoriasis treatment failure and the occurrence of severe infection.

## Methods

From January 2016 to December 2017, the authors consecutively included patients attending the Psoriasis Outpatient Clinic at the University Hospital of Brasília, University of Brasília, Brazil. This main reference hospital serves an urban area with more than 3 million inhabitants in Brasília Brazil. This strategy warranted internal validation of frequencies. Additional evaluations, including predictors and risk effects, were implemented according to a post hoc strategy.

### Inclusion criteria

The authors included patients with a diagnosis of moderate to severe psoriasis established through clinical assessment by two certified dermatologists. Whenever necessary, skin biopsies were performed. The authors excluded patients with a concomitant diagnosis of psoriatic arthritis or other autoimmune diseases.

### Baseline evaluation

All patients were subjected to a baseline evaluation comprised of a standardized form seeking demographic information, actual treatment information, and previous treatment information. Following the current literature and the international classification of diseases, patients who had the most common pattern of plaque psoriasis, associated or not with palmoplantar, nail, or scalp lesions were classified as having plaque psoriasis. Patients classified as scalp psoriasis, nail psoriasis, palmoplantar pustular psoriasis, guttate psoriasis, and inverse psoriasis had lesions exclusively in these locations. The medications used at the time of inclusion were considered basal medications. The Psoriasis Area and Severity Index (PASI), Body Surface Area (BSA), Dermatology Life Quality Index (DLQI), and Nail Psoriasis Severity Index (NAPSI) were evaluated whenever possible. Retrospective data regarding medication and disease history before inclusion presented important losses, and this information was removed from the outcome evaluation.

Total blood was collected by cubital venipuncture. Serum from each patient was isolated, and Interleukin-2 (IL-2), Interleukin-4 (IL-4), Interleukin-6 (IL-6), Interleukin-10 (IL-10), Interleukin-17A (IL-17A), Interferon (IFN)-γ and Tumor Necrosis Factor (TNF)-α were measured using the BD™ Cytometric Bead Array (CBA) Human Th1/Th2/Th17 Cytokine Kit (Becton Dickinson, Franklin Lakes, USA) with a FACSVerse flow cytometer (Becton Dickinson). All analyses were performed in triplicate, and the values were normalized and compared with those obtained from 47 healthy controls (mean age = 42 years and 3 months, 18 male and 29 female patients recruited at the same institution).

### Follow-up visits

Patients were followed up until December 2021 (up to 6 years) by a dermatologist through clinical consultation every 3 to 6 months.

### Primary endpoint

Drug survival was measured according to the time taken for basal medication interruption (change or suspension). The primary endpoint was considered the occurrence of drug interruption.^10^

### Secondary endpoints

As secondary endpoints, the authors considered the occurrences of treatment failure or severe infection. Treatment failure was defined as the necessity of treatment modification due to poor disease control (PASI, BSA, DLQI, and NAPSI scores above 10). Severe infection was defined as any infection that led to the suspension of clinical treatment for more than 1 month. The incidence of additional outcomes, such as adverse reactions, was also evaluated.

### Predictors

Relevant clinical and laboratory baseline characteristics that were assessed as candidate predictors of drug survival included sex, age, smoking habits, and alcohol abuse, as well as pro-inflammatory (IL-2, IL-6, IL-17A, IFN-γ TNF-α) and anti-inflammatory (IL-4, IL-10) cytokines. At baseline, patients that were on biologics only and who had never used other biologic drugs were classified as “naive”.

### Statistical analysis

Predictors were evaluated using log-rank tests. Data censoring was set to occur when patients were lost to follow-up. Numerical variables were not categorized in univariate analysis. For adjusted analysis, cytokine levels were categorized according to the median value of the global population or as positive/negative if more than 30% of the patients presented negative results. The remaining numerical variables were only categorized to generate graphics. PASI and BSA values were calculated only for patients with plaque psoriasis. Missing data were not replaced and patients with missing data were excluded from specific analyses.

For the primary outcome, the authors used an adjusted multivariate model. Predictors that met a low evidence threshold (p ≤ 0.100) based on univariate analysis were included. The authors calculated Hazard Ratios (HRs) using a Cox proportional hazards model. All assumptions were carefully evaluated. The authors used the survival and survminer packages in R Studio (R Studio, Vienna, Austria). For secondary outcomes, the authors used a subgroup analysis strategy. Graphical comparison of cytokine levels was performed using GraphPad Prism version 8.0.0 for Windows (GraphPad Software, San Diego). Statistical significance was defined by a p-value <0.05 and an appropriate 95% Confidence Interval (95% CI).

### Ethics

This study was approved by the Ethics Committee of the Faculty of Medicine of the University of Brasília. All patients were included after signing the informed consent form.

## Results

The authors followed up 262 patients with psoriasis; 133 (50.76%) were male, and 129 (49.24%) were female. Most patients presented with plaque psoriasis (n = 222; 84.73%). The authors also included patients with scalp psoriasis (n = 12; 4.58%), ungual psoriasis (n = 5; 1.91%), palmoplantar pustulous psoriasis (n = 8; 3.05%), guttate psoriasis (n = 13; 4.96%) and inverse psoriasis (n = 2; 0.76%) (Table 1).

**Table 1:** Univariate and multivariate analysis for predictors of drug interruption in the treatment of psoriasis.

Variable	Outcome		Total (n = 262)	p-value	p-value (log-rank test)	HR 95% CI (Cox proportional hazards regression)	p-value (Cox proportional hazards regression)
	Treatment interruption (n = 122)	No Treatment interruption (n = 140)					
**Sex**							
Male, n (%)	63 (51.64%)	70 (50.00%)	133 (50.76%)	0.888	0.760	‒	‒
Female, n (%)	59 (48.36%)	70 (50.00%)	129 (49.23%)				
Age: mean (SD)	48.17 (14.75)	52.79 (14.58)	50.64 (14.81)	0.012	‒	0.99 (0.98‒1.01)	0.302
Smoking habit n (%)	27 (22.13%)	19 (13.57%)	46 (17.56%)	0.104	0.063	1.29 (0.80‒2.08)	0.294
Alcohol abuse, n (%)	24(19.67%)	37 (26.43%)	61 (23.28%)	0.239	0.270	‒	‒
IL2 levels: median (IQR)	0.19 (2.36)	0.00 (1.46)		0.081	‒	1.03 (0.68‒1.56)	0.890^a^
IL4 levels: median (IQR)	0.53 (1.67)	0.12 (1.55)		0.255	‒	‒	‒
IL6 levels: median (IQR)	3.69 (3.83)	2.40 (3.27)		0.002	‒	1.99 (1.29‒3.08)	0.002^b^
IL10 levels: median (IQR)	1.11 (1.08)	0.97 (0.90)		0.312	‒	‒	‒
IL17A levels median (IQR)	3.13 (6.65)	1.44 (7.54)		0.344	‒	‒	‒
IFN levels: median (IQR)	0.30 (1.73)	0.20 (1.55)		0.478	‒	‒	‒
TNF levels: median (IQR)	0.10 (1.74)	0.04 (0.74)		0.335	‒	‒	‒
Associated medications, n (%)	5 (4.09%)	11 (7.86%)	16.00 (6.11%)	0.313	0.180	‒	‒
Previous therapeutic failures: median (IQR)	1.00 (1.75)	1.00 (1.25)	1.00 (1.75)	0.964	‒	‒	‒
Bioexclusive, n (%)	32 (26.23%)	36 (25.71%)	68 (25.95%)	1.000	0.760	‒	‒
**Psoriasis Classification**							
Plaques, n (%)	109 (89.34%)	113 (80.71%)	222 (84.73%)	0.462	0.810	‒	‒
Scalp n (%)	4 (3.28%)	8 (5.71%)	12 (4.58%)				
Ungual n (%)	1 (0.82%)	4 (2.86%)	5 (1.91%)				
Palmoplantar n (%)	2 (1.64%)	6 (4.29%)	8 (3.05%)				
Guttate n (%)	5 (4.10%)	8 (5.71%)	13 (4.96%)				
Inverse n (%)	1 (0.82%)	1 (0.71%)	2 (0.76%)				
Basal BSA mean (SD)	12.65 (18.71)	8.17 (16.00)		0.056	‒	‒	‒
Basal PASI mean (SD)	5.66 (6.58)	4.01 (6.02)		0.038	‒	0.99 (0.96‒1.03)	0.739
Basal DLQI mean (SD)	7.57 (7.79)	4.42 (6.10)		<0.001	‒	1.04 (1.01‒1.07)	0.012
Basal NAPSI mean (SD)	3.79 (7.76)	1.08 (3.50)		<0.001	‒	1.02 (0.99‒1.05)	0.113

BSA, Body Surface Area; PASI, Psoriasis Area and Severity Index; DLQI, Dermatology Life Quality Index.

a Cut-off set as a positive or negative result.

b Cut-off set as a value below, above, or equal to the median value of the global population.

In this study, 71 (27.10%) patients were not using systemic immunosuppressants [9 (3.44%) were without any treatment, 5 (1.91%) underwent isolated phototherapy, 50 (19.08%) used topical corticosteroids and 7 (2.67%) used acitretin]; 96 (36.64%) patients used classical immunosuppressors [94 (35.88%) used methotrexate and 2 (0.76%) used systemic corticosteroids]; 75 patients (28.63%) used anti-TNF biologics [22 (8.39%) used adalimumab, 29 (11.07%) used etanercept and 24 (9.16%) used infliximab]; and 20 patients (7.63%) used anti-IL drugs [9 (3.44%) used ustekinumab and 11 (4.19%) used secukinumab].

The mean follow-up time was 4.88 years (range = 1–6 years). Drug interruption before the end of the study follow-up was identified in 122 (46.56%) patients; 88 (33.58%) patients had an isolated treatment failure identified, and 18 (6.87%) patients had an isolated severe infection that led to treatment suspension. Sixteen patients (6.11%) had interrupted treatment because of simultaneous severe infection and treatment failure. In total, 34 (12.97%) patients presented with severe infections (25 patients had unidentified upper airway infections, 8 patients had COVID-19 diagnosis and 1 patient had a severe gastrointestinal infection). Additionally, the data of only 5 patients were censored (considered as a loss to follow-up) because of other reported adverse events. These events were only evaluated in a descriptive form because of the low number of events. One patient developed hidradenitis suppurative while on acitretin therapy, 2 patients presented with signs of neuropathy while using methotrexate, 1 patient presented with dyspnoea while using methotrexate, and 1 patient presented with signs of initial renal failure while using infliximab.

### Cytokine profile

**Figure 1:**
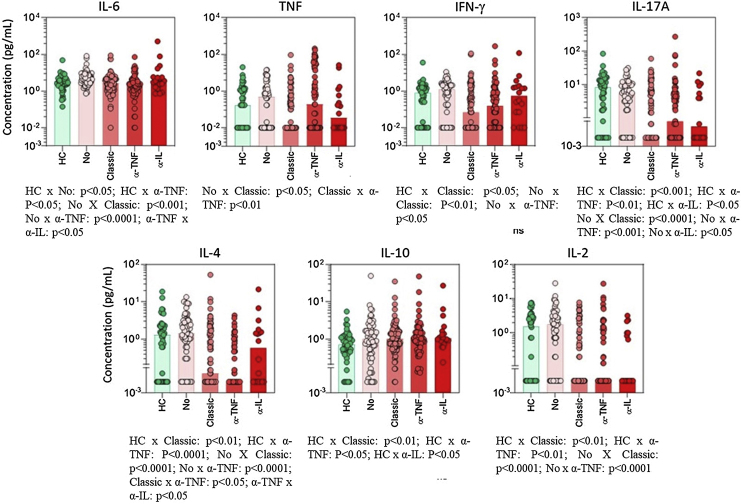
Serum cytokine levels in psoriasis patients according to the type of basal immunosuppressive therapy at the time of inclusion. Data are shown as a scattering distribution of individual values over bar charts representing the median levels of each cytokine. IL, Interleukin; TNF, Tumor Necrosis Factor; IFN, Interferon; HC, Healthy Controls; No, Psoriasis patients using no systemic immunosuppressors; Classic, Psoriasis patients using classic immunosuppressors; α-TNF, Psoriasis patients using anti-tumor necrosis factor agents; α-IL, Psoriasis patients using anti IL agents; p-values for all groups that presented a significant difference are disclosed at the bottom of each graphic. Non-significant p-values (> 0.05) are not represented.

**Figure 2:**
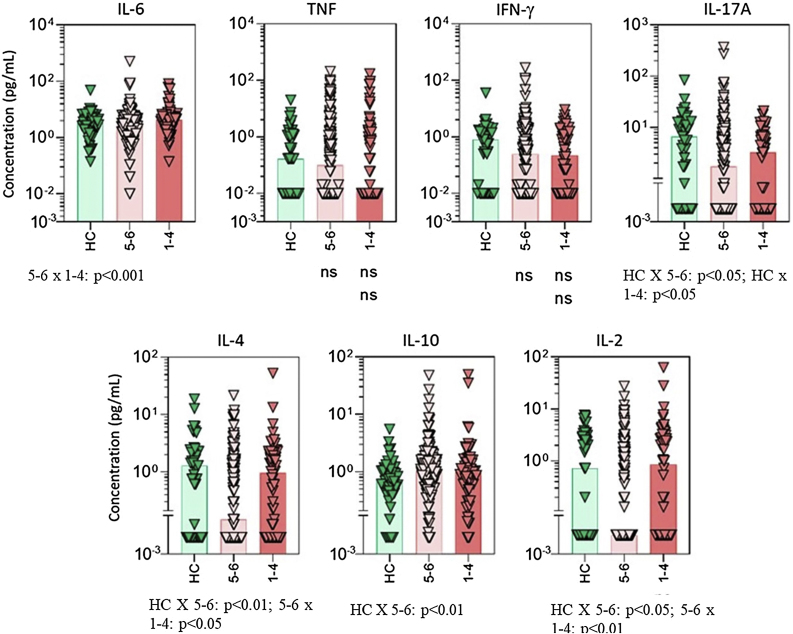
Serum cytokine levels in psoriasis patients according to drug survival stratified by 1 to 4 years and 5 to 6 years. Data are shown as scattering distribution of individual values over bar charts representing median levels of each cytokine. IL, Interleukin; TNF, Tumor Necrosis Factor; IFN, Interferon; HC, Healthy Controls; p-values for all groups that presented a significant difference are disclosed at the bottom of each graphic. Non-significant p-values (> 0.05) are not represented.

The use of classic immunosuppressants, mainly methotrexate, was associated with important suppression of proinflammatory cytokines (Fig. 1). For some mediators, such as IL-17 and TNF-α, the suppressive effect of methotrexate was even greater than the effect observed in patients using anti-TNF or anti-IL biologics. IL-6 baseline levels were significantly enhanced in patients with poorer survival rates (Fig. 2).

### Drug survival analysis

Univariate analysis (Table 1) showed that baseline PASI (p = 0.038), DLQI (p < 0.001), and NAPSI (p < 0.001) values were significantly greater in patients who interrupted systemic treatment. IL-6 was the only inflammatory marker that was significantly elevated in patients who had interruptions of their basal medication (p = 0.002).

**Figure 3:**
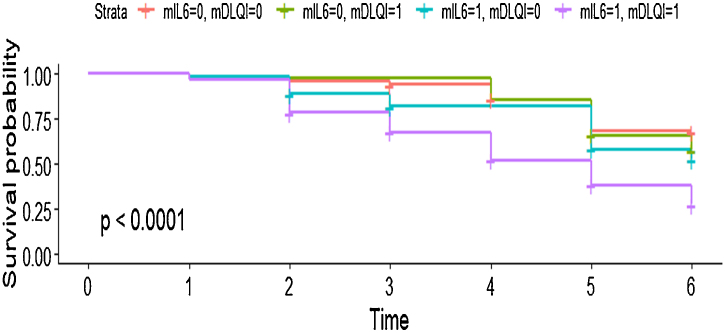
Survival curve showing the joint effect of the two main predictors of drug interruption Interleukin (IL)-6 levels and Dermatology Life Quality Index (DLQI). It can be seen that patients experiencing higher DLQI and IL-6 levels had an almost 75% chance of systemic treatment change in the follow-up period. mIL6, Median IL-6 scores of the present population (0 = below the median value; 1 = above the median value). mDLQI, DLQI score of the present population; 0 = below 5; 1 = above or equal 5.

In the multivariate model, the authors observed that each point elevation in the DLQI score elevated the chance of treatment change by 1.04 times with very narrow 95% CIs (HR = 1.04; 95% CI 1.01‒1.07; p = 0.012). Additionally, patients who had baseline IL-6 levels equal to or above the median value of the total population had an almost 2 times greater chance of interrupting systemic treatment than patients with lower levels of the proinflammatory marker (HR = 1.99; 95% CI 1.29‒3.08; p = 0.002) (Fig. 3). Detailed information related to the subgroup analysis can be found in Supplementary File 1, (Tables A and B).

## Discussion

Psoriasis is a complex inflammatory disease.^19^ It is didactic to consider that the beginning of the inflammatory cascade is jointly caused by stimuli of environmental factors associated with genetic susceptibility.^20^, ^21^ These stimuli activate antigen-presenting cells, macrophages and dendritic cells to produce coordinating cytokines (TNF, IL-23, IFN).^20^ Subsequently, the activation of the Th1 and Th17 pathways results in the activation of final cytokines, such as IL-17.^22^ Finally, inflamed keratinocytes also express inflammatory mediators.^19^

The utility of measuring cytokines in the blood of patients with skin conditions is under debate. Although most studies highlight the autocrine and paracrine effects of those mediators in psoriasis, virtually all involved cytokines have an endocrine-like effect.^23^, ^24^ Cataldi et al. found that plasma Th17 pathway mediators were significantly higher in a population of 70 psoriasis patients than in a control group.^25^

In the present study, IL-6 elevation was the most consistent proinflammatory marker for treatment interruption. Although IL-6 is a proinflammatory mediator, the use of tocilizumab, a recombinant humanized anti-IL-6 biologic, in psoriasis led to unpredictable results.^26^, ^27^ Studies that evaluated the inflammatory environment of the skin failed to include IL-6 expression in the main pathogenic pathway of psoriasis. Nakajima et al., in 2010, found that IL-6 was not crucial for the development of T-cell-independent psoriasis-like dermatitis in a murine model.^28^ The same result was found for IL-17, one of the main modern targets for psoriasis treatment, showing that the human inflammatory profile can be unpredictable.^28^

Interestingly, in line with the present findings, previous data that evaluated smaller populations reinforce the role of circulating IL-6 for the prediction of treatment response in psoriasis. Zalewska et al. found that plasma IL-6 levels were consistently elevated in 106 untreated psoriasis patients and that elevated levels of these mediators were related to poor treatment response.^29^ In addition, several studies have related the levels of circulating IL-6 to the systemic inflammation profile of psoriasis patients, including the occurrence of psoriatic arthritis and dyslipidemia.^30^, ^31^, ^32^, ^33^, ^34^, ^35^, ^36^, ^37^

It is possible that IL-6 is not very important for triggering the inflammatory cascade of psoriasis in the skin environment, but its suppressive effect on regulatory T-cells may explain why this mediator is a more important marker for treatment response.^38^ Additionally, previous studies have shown that IL-6 is one of the inflammatory mediators with the most important systemic effects.^23^ Joint modulation of IL-6 and other cytokines, as achieved by Janus kinase inhibitors, may be an interesting strategy for refractory cases, as demonstrated by phase 2 clinical trials.^39^

Classic medications, mainly methotrexate, were associated with lower levels of TNF and IL-17 (Figure 1, Figure 2). Considering that methotrexate is clinically less effective than biologics, the authors hypothesize that an intense suppression of the two main mediators for psoriasis is not directly correlated to treatment responses. On the other hand, patients responding to methotrexate may present with milder cases of psoriasis and, consequently, have lower levels of TNF and IL-17. Unfortunately, in the present study, the authors did not measure inflammatory cytokines at different time points. Long-term serial evaluation of those markers can offer better clues related to the pathogenesis of psoriasis.

Additionally, in the present study, poor scores of traditional clinical markers, including PASI, DLQI, and NAPSI, were significantly related to reduced drug survival. This result was expected since these parameters are internationally recognized predictors of drug switching. Quality of life (DLQI) had the greatest influence on drug survival among the evaluated clinical scores. The main limitation of any cohort study is the presence of confounders that cannot be treated by randomization. However, the IL-6 results were consistent in the laboratory analysis, the univariate analysis, and the multivariate model. This result also represents a reproduction of previous studies in smaller populations.^31^ Furthermore, the authors only aimed to measure baseline cytokine levels as long-term markers for psoriasis severity. Multiple measurements at different time points may help to elucidate the role of serum markers in the evolution of psoriasis and its treatment.

## Conclusion

The present study showed that reduced quality of life and elevated serum IL-6 levels were predictors of poor drug survival in patients with moderate to severe psoriasis. The authors can also conclude that although IL-6 is not the most important mediator of the inflammatory pathway in the skin environment, it is an interesting candidate biomarker for predicting psoriasis systemic treatment response.

## Financial support

The research was funded by Fundação de Apoio à Pesquisa do Distrito Federal (FAP-DF) Grant numbers: FINATEC 7044 and 7155; and by La Roche Posay Foundation.

## Authors' contributions

Natália Ribeiro de Magalhães Alves: Investigation, writing ‒ original draft, writing ‒ review & editing.

Patrícia Shu Kurizky: Investigation, writing ‒ original draft, writing ‒ review & editing, project administration, supervision.

Licia Maria Henrique da Mota: Investigation, writing ‒ review & editing, supervision.

Cleandro Pires de Albuquerque: Investigation, writing ‒ review & editing.

Juliana Tomaz Esper: Investigation.

Aridne Souza Costa Campos: Investigation.

Vitoria Pereira Reis: Investigation.

Henrique Metzker Ferro: Investigation.

Natalia Gil-Jaramillo: Investigation.

Joaquim Pedro Brito-de-Sousa: Investigation.

Luana Cabral Leao Leal: Investigation.

Otávio de Toledo Nóbrega: Investigation, writing ‒ review & editing, supervision.

Carla Nunes de Araújo: Investigation, writing ‒ original draft, supervision.

Agenor de Castro Moreira dos Santos Júnior: Investigation, writing ‒ original draft.

Gladys Aires Martins: Investigation, supervision.

Olindo Assis Martins Filho: Investigation, writing ‒ original draft, writing ‒ review & editing, supervision.

Ciro Martins Gomes: Investigation, writing ‒ review & editing, project administration, funding acquisition.

## Conflicts of interest

None declared.

## References

[1] Huerta C., Rivero E., Rodríguez L.A.G. (2007). Incidence and risk factors for psoriasis in the general population. Arch Dermatol..

[2] Menter A., Strober B.E., Kaplan D.H., Kivelevitch D., Prater E.F., Stoff B. (2019). Joint AAD-NPF guidelines of care for the management and treatment of psoriasis with biologics. J Am Acad Dermatol..

[3] Armstrong A.W., Puig L., Joshi A., Skup M., Williams D., Li J. (2020). Comparison of biologics and oral treatments for plaque poriasis: a meta-analysis. JAMA Dermatol..

[4] Sbidian E., Chaimani A., Afach S., Doney L., Dressler C., Hua C. (2020). Systemic pharmacological treatments for chronic plaque psoriasis: a network meta-analysis. Cochrane Database Syst Rev..

[5] Mahil S.K., Smith C.H. (2019). Psoriasis biologics: a new era of choice. Lancet..

[6] Bellinato F., Gisondi P., Girolomoni G. (2021). Latest advances for the treatment of chronic plaque psoriasis with biologics and oral small molecules. Biologics..

[7] Schmitt-Egenolf M. (2006). Psoriasis therapy in real life: the need for registries. Dermatology..

[8] Chen Y.C., Huang Y.T., Yang C.C., Lai E.C.C., Liu C.H., Hsu C.K. (2020). Real-world efficacy of biological agents in moderate-to-severe plaque psoriasis: An analysis of 75 patients in Taiwan. PLoS One..

[9] Eissing L., Rustenbach S.J., Krensel M., Zander N., Spehr C., Radtke M.A. (2016). Psoriasis registries worldwide: systematic overview on registry publications. J Eur Acad Dermatol Venereol..

[10] Lima E.C., Boza J.C., Palominos P.E., Xavier R.M., Cestari T.F. (2021). Survival of immunobiological drugs in psoriasis: preliminary data from a Tertiary Hospital experience in Southern Brazil. An Bras Dermatol..

[11] Boehncke W.H. (2018). Systemic Inflammation and Cardiovascular Comorbidity in Psoriasis Patients: Causes and Consequences. Front Immunol..

[12] Korman N.J. (2020). Management of psoriasis as a systemic disease: what is the evidence?. Br J Dermatol..

[13] Christophers E., van de Kerkhof P.C.M. (2019). Severity, heterogeneity, and systemic inflammation in psoriasis. J Eur Acad Dermatol Venereol..

[14] Kurizky P.S., Martins G.A., Carneiro J.N., Gomes C.M., Mota L.M.H. (2018). Evaluation of the occurrence of sexual dysfunction and general quality of life in female patients with psoriasis. An Bras Dermatol..

[15] Olsson S., Pal S.N., Stergachis A., Couper M. (2010). Pharmacovigilance activities in 55 low- and middle-income countries: a questionnaire-based analysis. Drug Saf..

[16] Lupatini E.O., Zimmermann I.R., Barreto J.O.M., Silva E.N. (2022). How long does it take to translate research findings into routine healthcare practice? ‒ the case of biological drugs for rheumatoid arthritis in Brazil. Ann Transl Med..

[17] Romiti R., Fabrício L.H.Z., Souza C.S., Galvão L.O., Castro C.C.S., Terena A.C. (2018). Assessment of psoriasis severity in Brazilian patients with chronic plaque psoriasis attending outpatient clinics: a multicenter, population-based cross-sectional study (APPISOT). J Dermatolog Treat..

[18] antigo-conitec [Internet]. Brasil. Ministério da Saúde do Brasil. Protocolo Clínico e Diretrizes Terapêuticas da Psoríase. [cited 2023 Feb 8]. Available from: http://antigo-conitec.saude.gov.br/images/Relatorios/2021/20211021_Relatorio_652_PCDT_Psoriase.pdf.

[19] Ni X., Lai Y. (2020). Keratinocyte: a trigger or an executor of psoriasis?. J Leukoc Biol..

[20] Ayroldi E., Bastianelli A., Cannarile L., Petrillo M.G., Delfino D.V., Fierabracci A. (2011). A pathogenetic approach to autoimmune skin disease therapy: psoriasis and biological drugs, unresolved issues, and future directions. Curr Pharm Des..

[21] Reich K. (2012). The concept of psoriasis as a systemic inflammation: Implications for disease management. J Eur Acad Dermatol Venereol..

[22] Furiati S.C., Catarino J.S., Silva M.V., Silva R.F., Estevam R.B., Teodoro R.B. (2019). Th1, Th17, and Treg responses are differently modulated by TNF-α inhibitors and methotrexate in psoriasis patients. Sci Rep..

[23] Papanicolaou D.A., Vgontzas A.N. (2000). Interleukin-6: the endocrine cytokine. J Clin Endocrinol Metab..

[24] Miossec P. (2021). Local and systemic effects of IL-17 in joint inflammation: a historical perspective from discovery to targeting. Cell Mol Immunol..

[25] Cataldi C., Mari N.L., Lozovoy M.A.B., Martins L.M.M., Reiche E.M.V., Maes M. (2019). Proinflammatory and anti-inflammatory cytokine profiles in psoriasis: use as laboratory biomarkers and disease predictors. Inflamm Res..

[26] Hughes M., Chinoy H. (2013). Successful use of tocilizumab in a patient with psoriatic arthritis. Rheumatology..

[27] Hayakawa M., Izumi K., Higashida-Konishi M., Ushikubo M., Tsukamoto M., Akiya K. (2019). Tocilizumab-induced psoriasis-like eruption resolved by shortening the dose interval in a patient with rheumatoid arthritis: a case-based review. Rheumatol Int..

[28] Nakajima A., Matsuki T., Komine M., Asahina A., Horai R., Nakae S. (2010). TNF, but not IL-6 and IL-17, is crucial for the development of T cell-independent psoriasis-like dermatitis in Il1rn-/- mice. J Immunol..

[29] Zalewska A., Głowacka E., Wyczółkowska J., Tchórzewski H., Narbutt J., Sysa-Jedrzejowska A. (2006). Interleukin 6 and 8 levels in plasma and fibroblast cultures in psoriasis. Mediators Inflamm..

[30] Muramatsu S., Kubo R., Nishida E., Morita A. (2017). Serum interleukin-6 levels in response to biologic treatment in patients with psoriasis. Mod Rheumatol..

[31] Pietrzak A., Chabros P., Grywalska E., Pietrzak D., Kandzierski G., Wawrzycki B.O. (2020). Serum concentration of interleukin 6 is related to inflammation and dyslipidemia in patients with psoriasis. Postepy Dermatol Alergol..

[32] Klebow S., Hahn M., Nikoalev A., Wunderlich F.T., Hövelmeyer N., Karbach S.H. (2016). IL-6 signaling in myelomonocytic cells is not crucial for the development of IMQ-induced psoriasis. PLoS One..

[33] Tanaka R., Ichimura Y., Kubota N., Saito A., Nakamura Y., Ishitsuka Y. (2020). Activation of CD8 T cells accelerates anti-PD-1 antibody-induced psoriasis-like dermatitis through IL-6. Commun Biol..

[34] Sobolev V.V., Denisova E.V., Chebysheva S.N., Geppe N.A., Korsunskaya I.M. (2022). IL-6 gene expression as a marker of pathological state in psoriasis and psoriatic arthritis. Bull Exp Biol Med..

[35] Fujishima S., Watanabe H., Kawaguchi M., Suzuki T., Matsukura S., Homma T. (2010). Involvement of IL-17F via the induction of IL-6 in psoriasis. Arch Dermatol Res..

[36] Xu H., Liu J., Niu M., Song S., Wei L., Chen G. (2021). Soluble IL-6R-mediated IL-6 trans-signaling activation contributes to the pathological development of psoriasis. J Mol Med..

[37] Saggini A., Chimenti S., Chiricozzi A. (2014). IL-6 as a druggable target in psoriasis: focus on pustular variants. J Immunol Res..

[38] Goodman W.A., Levine A.D., Massari J.V., Sugiyama H., McCormick T.S., Cooper K.D. (2009). IL-6 signaling in psoriasis prevents immune suppression by regulatory T cells. J Immunol..

[39] Krueger J., Clark J.D., Suárez-Fariñas M., Fuentes-Duculan J., Cueto I., Wang C.Q. (2016). Tofacitinib attenuates pathologic immune pathways in patients with psoriasis: a randomized phase 2 study. J Allergy Clin Immunol..

