# Supplementary material for: Elevated serum IL-6 levels predict treatment interruption in patients with moderate to severe psoriasis: a 6-year real-world cohort study
Source: An Bras Dermatol. 2023 Aug 25;99(1):34–42. doi: 10.1016/j.abd.2023.03.002 (PMC10964352; doi:10.1016/j.abd.2023.03.002)
Supplement: Supplementary file 1 [file mmc1.docx]

**ABD-D-22-00568_ Supplementary Material**

**Supplementary File 1 Subgroup analysis of different outcomes (therapeutic failure and severe infection) according to the basal treatment.**

**Table A** The frequency and types of drugs used and the proportion of patients who experienced therapeutic failure.

| **Baseline drug** | **Treatment failure** | **No treatment failure** | **Total** | **Bioexclusive patients** |
| --- | --- | --- | --- | --- |
| No treatment | 1 | 8 | 9 | ‒ |
| Phototherapy | 1 | 4 | 5 | ‒ |
| Topical steroids | 25 | 25 | 50 | ‒ |
| Acitretin | 2 | 5 | 7 | ‒ |
| Methotrexate | 37 | 57 | 94 | ‒ |
| Systemic Steroids | 1 | 1 | 2 | ‒ |
| Adalimumab | 9 | 13 | 22 | 18 |
| Etanercept | 13 | 16 | 29 | 21 |
| Infliximab | 7 | 17 | 24 | 23 |
| Ustequinumab | 3 | 6 | 9 | 3 |
| Secuquinumab | 5 | 6 | 11 | 3 |
| No immunossupression | 29 | 42 | 71 | ‒ |
| Classic Immunosuppressors | 38 | 58 | 96 | ‒ |
| Anti-TNF | 29 | 46 | 75 | 62 |
| Anti-Interleukin Drugs | 8 | 12 | 20 | 6 |

**Table B** The frequency and types of drugs used and the proportion of patients who experienced severe infections.

| **Baseline drug** | **Severe infection** | **No severe infection** | **Total** |
| --- | --- | --- | --- |
| No treatment | 0 | 9 | 9 |
| Phototherapy | 1 | 4 | 5 |
| Topical steroids | 5 | 45 | 50 |
| Acitretin | 2 | 5 | 7 |
| Methotrexate | 14 | 80 | 94 |
| Systemic Steroids | 0 | 2 | 2 |
| Adalimumab | 2 | 20 | 22 |
| Etanercept | 2 | 27 | 29 |
| Infliximab | 5 | 19 | 24 |
| Ustequinumab | 1 | 8 | 9 |
| Secuquinumab | 2 | 9 | 11 |
| No immunossupression | 8 | 63 | 71 |
| Classic Immunosuppressors | 14 | 82 | 96 |
| Anti-TNF | 9 | 66 | 75 |
| Anti-Interleukin Drugs | 3 | 17 | 20 |
